# New Hospitals v. Hospital Extension for London

**Published:** 1894-02-10

**Authors:** 


### The Institutional Workshop.

### HOSPITAL ADMINISTRATION.

### NEW HOSPITALS *VERSUS* HOSPITAL EXTENSION FOR LONDON.

II.—Station Hospitals.

Last week we dealt fully with the considerations affecting the question of additional beds at the existing metropolitan hospitals, and intimated to what extent new hospitals in outlying districts, where such accommodation is needed, ought properly to be supplied. To-day we have to consider how far and to what extent it may be possible (2) to meet the needs of the sick, non-pauper poor, whilst offering safeguards against the risks and dangers which we fully indicated in our previous article. See The Hospital of the 3rd inst., page 317. Mr. Burdett, in his evidence before the Lords' Committee, laid stress upon the importance of establishing outpost hospitals, or receiving wards, in connection with the principal general hospitals in all large towns, and especially in London. The only objection which has been urged to this proposal is that it might unduly interfere with the work of the medical school. A close examination of the experience gained at the branch hospital, in connection with the "Dreadnought," and a due consideration of this aspect of the question under all its bearings, leads us to conclude that in practice there is nothing material in this criticism. Unless, or until one great medical school or university is established for the whole of London, an outpost hospital would materially aid the teachers in the medical schools, because it would ensure that many important cases, which do not at present come to the hospital, would be received at the outpost institution, and so in due course find their way into the clinical wards. A most interesting report on this subject was issued at the end of 1892 by the trustees of the Boston City Hospital, Mass. We have not space to do more than summarise its conclusions.

After two years' careful investigation the trustees considered the question under three aspects for each district of Boston; first, the small but complete general hospital; secondly, the emergency hospital, where patients should be temporarily received and treated, and when necessary sent forward to the parent hospital; thirdly, a hospital station for imparting first aid to the sick and injured, and assisting them to reach the central hospital. In the result the trustees decided that any local benefit which might arise from the establishment of local hospitals in outlying districts would not be sufficient to offset their disadvantages. Secondly, the trustees seem to have been frightened by the evidence of the managers of the emergency hospitals of New York, who declare the management to be attended by great difficulties and annoyances, owing to the tendency of the local staff to use the emergency hospitals as schools of medical observation, and to retain through favouritism, or for other reasons, cases which should be transferred to the central hospital. This objection would not, however, apply to outpost hospitals in London, if they were placed under the management of the authorities of the existing hospitals. Thirdly, the trustees, appreciating the fact that the inhabitants of the outlying districts of Boston, like those of London, are at considerable disadvantage as to hospital accommodation as compared with others in the central portion of the city, recommended the extension of the ambulance system, and the establishment of a hospital station in each district, which shall be in connection with the ambulance, and to which accident or emergency cases may be taken for immediate treatment, with a view to permanent provision. Each station is to be provided with two or three beds, all suitable appliances, and a trained nurse to care for and afford to the sufferers the necessary first aid. Daily one of the admittance staff of the mother hospital will attend to investigate applications for admission to it. The local relief staff consists of two local medical men, attached to each hospital station, with which their residences will be connected by telephone. It is in contemplation to ultimately appoint a skilled house officer from the City Hospital for service at each station, with a possible provision for out-patients, the ambulance work being done by the members of the police department. The control and conduct of the station hospitals will be committed to the Boston City Hospital authorities, *i.e.*, to the parent institution. One station hospital has been already opened as an experiment, but the results have not yet been made public. It is estimated that each station hospital will cost from £600 to £1,000 initially for fittings and furniture, and £1,000 a year to maintain. We are inclined to agree with the conclusions of the Boston trustees that "unnecessarily to divide hospitals is retrogression, and that to strengthen and concentrate hospital treatment is progress, in what most vitally concerns the well-being of every citizen."

To sum up the whole matter, it appears, then, that the existing metropolitan hospitals will be wise to keep the control of hospital treatment in their own hands by establishing under their management outpost station hospitals—*i.e.*, to open a few of each type experimentally in selected districts within the seven miles radius. The twelve metropolitan general hospitals with medical schools have an income of upwards of £190,000 a year from invested property, and are, therefore, strong enough financially to undertake this work if they think well to do so. Failing such action on their part, the time is approaching when the inhabitants of each district will take the matter into their own hands, as the people of North London have done, and with or without the aid of the London County Council, proceed to establish local hospitals like those at Greenwich and West Ham.

We see no reasonable objection that can be urged to the immediate erection of a large general hospital in South-East London, where the bed accommodation is lamentably deficient, especially as there is evidence to show that the absence of such provision has made the million and a half of people inhabiting that district singularly apathetic to the claims of the hospitals, which receive comparatively little money from them at the present time. But we do see many and grave objections to the spread of such a movement all over London, and we therefore commend this pressing question, in all its bearings, to the immediate attention of the authorities of the existing voluntary hospitals of London, and especially to those which have medical schools attached. Until the margin of 1,200 vacant beds now available disappears there is, of course, time for deliberation and experiment. Still, London is increasing at such a ratio, that the great hospitals will be wise to formulate a carefully considered plan of action without delay, and so make themselves masters of the situation in self-defence.

